# Annual Report of the Connecticut School for Imbeciles

**Published:** 1872-10

**Authors:** 


*Annual Report of the Connecticut School for Imbeciles*, at Lakeville, Conn. 1872.

The report shows this school in a prosperous condition, producing as much good among this unfortunate class as the limited means afforded it will allow An appeal has been made to the State for further aid, which we hope will meet a speedy response. The State should take as tender care of this class of unfortunates as it does of the insane.

The school is under the superintendence of Dr. H. M. Knight, who is eminently fitted for the place.

